# Efficient and Convenient Synthesis of 1,8-Dioxodecahydroacridine Derivatives Using Cu-Doped ZnO Nanocrystalline Powder as a Catalyst under Solvent-Free Conditions

**DOI:** 10.1155/2013/575636

**Published:** 2013-10-30

**Authors:** Heshmatollah Alinezhad, Sahar Mohseni Tavakkoli

**Affiliations:** Faculty of Chemistry, University of Mazandaran, Babolsar, Iran

## Abstract

A simple and convenient one-step method for synthesis of acridines and their derivatives from condensation of aromatic aldehydes, cyclic diketones, and aryl amines using Cu-doped ZnO nanocrystalline powder as a catalyst is reported. The present protocol provides several advantages such as good yields, short reaction time, easy workup, and simplicity in operation.

## 1. Introduction

In recent years, an increasing interest has been focused on the synthesis of 1,4-dihydropyridine compounds owing to their significant biological activities [1]. Substituted acridines have been used as antimalarials [2] for many years quite successfully and several of them have exhibited excellent results in chemotherapy of cancer [3–6]. These derivatives are frequently used in industry, especially for the production of dyes [7, 8]. Beside these properties, analogues of acridine have also been shown to have very long lasting efficiencies and have interesting electrochemical behavior [9, 10] of heterocyclic compounds and in the interaction with DNA [11].

Some methods are available in the literature for the synthesis of acridine derivatives containing 1,4-dihydropyridines, from dimedone, aldehyde, and different nitrogen sources like urea [12], methyl amine [13], and different anilines or ammonium acetate [14] via traditional heating in organic solvents in the presence of triethylbenzylammonium chloride (TEBAC) [15], *p*-dodecylbenzenesulfonic acid (DBSA) [16], Proline [17], Amberlyst-15 [18], ammonium chloride, Zn(OAc)_2_·H_2_O or L-proline [19], and/or under solvent-free conditions such as under microwave irradiation [20–22], sulfonic acid functionalized silica [23], ZnO nanoparticles [24], and nano-Fe_3_O_4_ [25] and using ionic liquids [26, 27] such as 1-methylimidazolium trifluoroacetate ([Hmim]TFA) [28] and Bronsted acidic imidazolium salts containing perfluoroalkyl tails [29]. Furthermore, some of these procedures suffer from other disadvantages, including the requirement for an expensive catalyst or the use of an excess of catalyst. To avoid these limitations and to improve the reaction conditions available for the synthesis of 1,8-dioxodecahydroacridines, the discovery of new methodologies using new heterogeneous and reusable catalysts is still in demand.

ZnO is considered to be one of the most important oxide materials owing to its unique features and wide range of technologically important applications. Moreover,it is cheap and environmentally friendly as compared to other metal oxides. Due to these properties, it has found potential applications in several fields such as gas sensors [30], solar cells [31], varistors [32], light emitting devices [33], photocatalyst [34], antibacterial activity [35], and cancer treatment [36]. ZnO lacks center of symmetry, which makes it beneficial for use in actuators and piezoelectric transducers. Properties of ZnO can be tuned according to the research interest, by doping with various metal atoms to suit specific needs and applications. The metal doping induces drastic changes in optical, electrical, and magnetic properties of ZnO by altering its electronic structure. Many authors have reported the changes induced by incorporation of transition metal ions into ZnO lattice [37–39]. albeit the large number of reports on transition metal-doped ZnO system, very less work is done on Cu-doped ZnO. Substitution of copper into the ZnO lattice has been shown to improve properties such as photocatalytic activity, gas sensitivity, and magnetic semiconductivity [40–42].

**Scheme 1:**
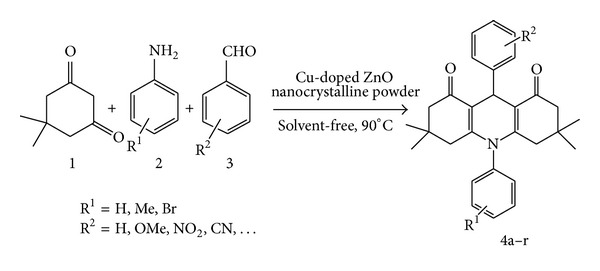
The synthesis of 1,8-dioxodecahydroacridines in solvent-free conditions.

In continuation of our studies in developing efficient, simple, and environmentally benign methodologies for organic synthesis, we reveal herein the synthesis of *N*-substituted decahydroacridine-1,8-diones using Cu-doped ZnO nanocrystalline powder as a catalyst under solvent-free condition (Scheme 1).

## 2. Materials and Methods

### 2.1. General

All materials were purchased from Merck. The reactions were monitored by TLC using silica gel plates, and the products were purified by flash column chromatography on silica gel (Merck, 230–400 mesh) and were identified by comparison of their spectra (^1^HNMR and ^13^CNMR) and physical data with those of the authentic samples. ^1^H NMR and ^13^C NMR spectra were recorded with Brucker DRX500 AVANCE (400 MHz) spectrometers, using CDCl_3_ as solvent. The morphology and elemental composition were characterized by a digital microscopy imaging scanning equipment VEGA 3 SB (TESCAN Co., s.r.o., Brno, Czech Republic) and energy dispersive X-ray spectrometer (EDS) attached to the SEM instrument with the operating voltage of 15 kV.

### 2.2. Synthesis of Cu-Doped ZnO Nanocrystalline Powder

Synthesis of Zn_1−*x*_Cu_*x*_O (1% Cu-doped) nanopowder was carried out using the same technique followed by Cadar et al. [43] with some modifications after optimization of reaction conditions. The targets in the experiment were specifically designed using high purity of zinc nitrate hexahydrate (99.99%) and copper sulfate pentahydrate (99%) powders. The copper-doped ZnO catalyst was prepared by a two-step procedure: (1) preparation of precursor by coprecipitation method; (2) formation of Cu/ZnO nanopowder by thermal decomposition. The method has been considered to be fast, simple, and inexpensive, allowing for the production of fine, homogeneous crystalline powders without the risk of contamination.

The stoichiometric quantities of zinc and copper salts were dissolved in 100 mL deionized double distilled water (solution A). Separately, a solution was prepared by dissolving appropriate amounts of sodium hydroxide and sodium carbonate in deionized double distilled water (solution B). The solution A was heated to 85°C and the solution B was mixed dropwise into this solution with constant stirring. During the whole process, temperature was maintained at 85°C. This mixing was done for 1 h while refluxing through water condenser 85°C. Final solution was allowed to cool at room temperature and greenish precipitate that formed was washed three times with 20 mL deionized water in order to remove unnecessary impurities and dried overnight at 50°C under vacuum. Finally, the precursors were calcined at a temperature of 450°C for 90 min in the muffle furnace under air atmosphere to obtain the nanocrystalline Cu/ZnO powder.

**Figure 1:**
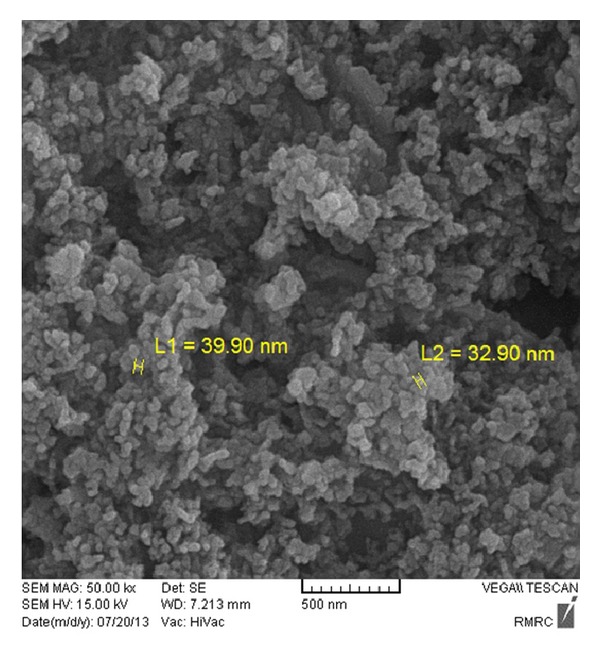
SEM photo of 1% Cu-doped ZnO nanocrystalline powder.

**Figure 2:**
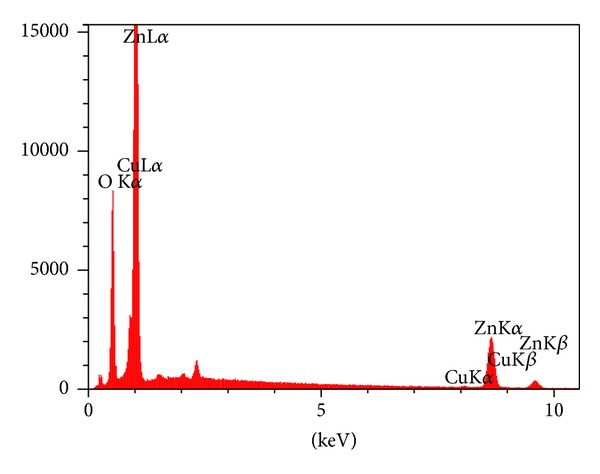
Representative EDX spectra of 1% Cu-doped nanocatalyst sample.

Figure 1 shows scanning electron micrograph of nanocrystalline Zn_1−*x*_Cu_*x*_O sample. In order to confirm the presence of Cu in the synthesized ZnO nanoparticles, the compositional analysis and purity of the as-synthesized nanocatalyst was obtained using EDS.  Figure 2 shows the representative EDX spectra of nanocrystalline sample that the estimated amount of Cu dopant was nearly 1%. From the similarity of the Zn and Cu peak intensity line traces, it is clear that after the synthesis process, zinc and copper were homogenously distributed inside the nanoparticle.

### 2.3. General Experimental Procedure for *N*-Substituted Decahydroacridine-1,8-diones Formation Catalyzed by Cu-Doped ZnO Nanocrystalline Powder (Table 3, Entry 1)

A mixture of dimedone 1 (2 mmol), aromatic amine 2 (1 mmol), aromatic benzaldehyde 3 (1 mmol), and 10 mol% of Cu-doped ZnO nanocrystalline powder was heated in an oil bath at 90°C for 1.5 or 2 h. The reaction process was monitored by TLC (n-Hexane: EtOAc, 1 : 1). Upon completion of the transformation, the reaction mixture was cooled to room temperature and hot ethanol (15 mL) was added. This resulted in the precipitation of the catalyst, which was collected by filtration. The filtrate was distilled to dryness to give the crude product, which was recrystallized from a mixture of EtOH and H_2_O to give compounds 4 in high to excellent yields (Scheme 1).

## 3. Results and Discussion

At first, to optimize the reaction condition, we studied the reaction of dimedone (2 mmol), aniline (1 mmol), and benzaldehyde (1 mmol) as model compounds in the presence of Cu-doped ZnO nanocrystalline powder (10 mol%) as a catalyst in solvent-free condition. We evaluated the effect of different solvents such as ethanol, H_2_O, DMF, CH_2_Cl_2_, and solvent-free condition on the reaction rate under the same reaction conditions. Solvent-free condition afforded the products in higher yield and shorter reaction time (Table 1).

We next investigated the other amounts of Cu-doped ZnO nanocrystalline powder (5 and 15 mol%) for this reaction. The optimum yield of the *N*-substituted decahydroacridine-1,8-diones was obtained when 10 mol% of Cu-doped ZnO nanocrystalline powder was used (Table 2).

Therefore, in an optimized reaction condition dimedone (2 mmol), amine (1 mmol), and benzaldehyde (1 mmol) in an oil bath at 90°C were mixed with Cu-doped ZnO nanocrystalline powder (10 mol%) for 1.5 or 2 h (Table 3).

Subsequently, a variety of *N*-substituted decahydroacridine-1,8-diones were prepared from dimedone, various benzaldehyde derivatives, aniline derivatives, and benzylamine using the optimized reaction conditions and the results are summarized in Table 3.

As shown in Table 3 when the electron withdrawing substituents are present in benzaldehyde, the reaction rate increases, whereas the effect is reversed in the case of benzaldehyde with strong electron-donating substituents such as –OMe and –OH, of course with lower yields (entries 2, 13, 14, and 15). Orthosubstituted benzaldehyde also required relatively long reaction time towards para- and metasubstituted benzaldehydes  (entries 7, 8, 9), because of its steric effect. 

**Scheme 2:**
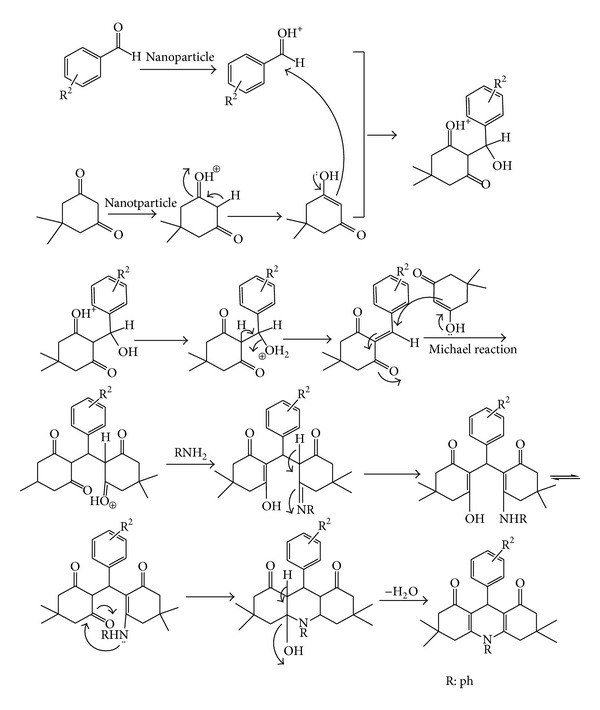
The proposed mechanism of synthesis of 1,8-dioxodecahydroacridines.

A plausible mechanism for the formation of the 1,8-dioxodecahydroacridine products using Cu-doped ZnO nanocrystalline powder as a catalyst has been depicted in Scheme 2. We propose that the nanoparticle induces the polarization of the carbonyl groups and facilitates the formation of the intermediates that subsequently react together to give the final products 4a–4r.

## 4. Conclusion

We have developed a new catalytic method for synthesis of 1,8-dioxodecahydroacridines using Cu-doped ZnO nanocrystalline powder as a catalyst in solvent-free condition by one-pot three-component condensation of aromatic aldehydes, dimedone, and aromatic and aliphatic amines. The simple experimental, workup procedure and catalyst preparation, high to excellent yields and using catalytic amount of Cu-doped ZnO nanocrystalline powder, are notable advantages of the method.

## Figures and Tables

**Table 1:** Optimization of the reaction conditions^a^.

Entry	Solvent	Time (h)	Yield (%)^b^
1	Ethanol	6	30
2	H_2_O	5	30
3	CH_2_Cl_2_	4	20
4	DMF	6	40
5	—	1.5	90

^a^Reactions were carried out with dimedone, aniline, and benzaldehyde in 2 : 1 : 1 molar ratio.

^b^Yields refer to isolated pure products.

**Table 2:** Effect of amount of catalyst on the synthesis of* N*-substituted Decahydroacridine-1,8-diones^a^.

Entry	Catalyst (mol%)	Time (h)	Yield (%)^b^
1	5	3.5	90
2	10	1.5	90
3	15	1.5	90

^a^Reactions were carried out with dimedone, aniline, and benzaldehyde with molar ratio of 2 : 1 : 1.

^b^Yields refer to isolated pure products.

**Table 3:** Synthesis of *N*-substituted decahydroacridine-1,8-diones using Cu-doped ZnO nanocrystalline powder (10 mol%) as a catalyst in solvent-free condition^a^.

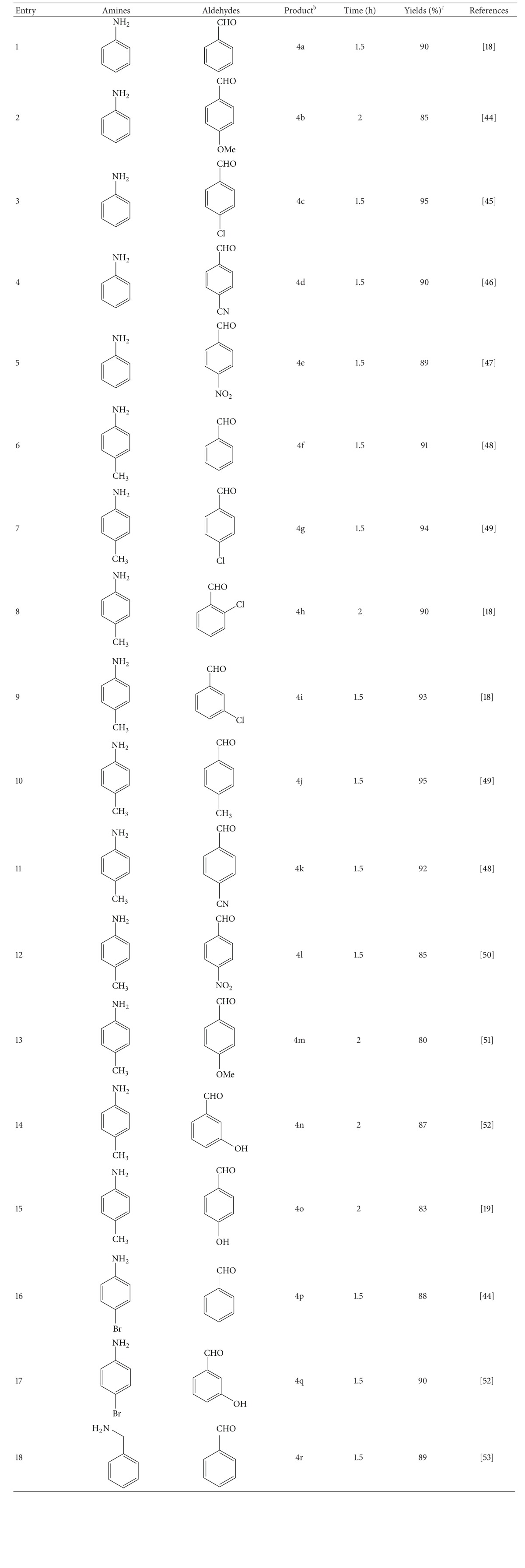

^a^Reactions were carried out with dimedone, amine, and benzaldehyde in 2 : 1 : 1 molar ratio.

^b^products were characterized with ^1^H, ^13^C-NMR, mp.

^c^Yields refer to isolated pure products.
